# Supplementary figures and images for: Monocyte-secreted Wnt reduces the efficiency of central nervous system remyelination
Source: PLoS Biol. 2025 Apr 15;23(4):e3003073. doi: 10.1371/journal.pbio.3003073 (PMC12052099; doi:10.1371/journal.pbio.3003073)

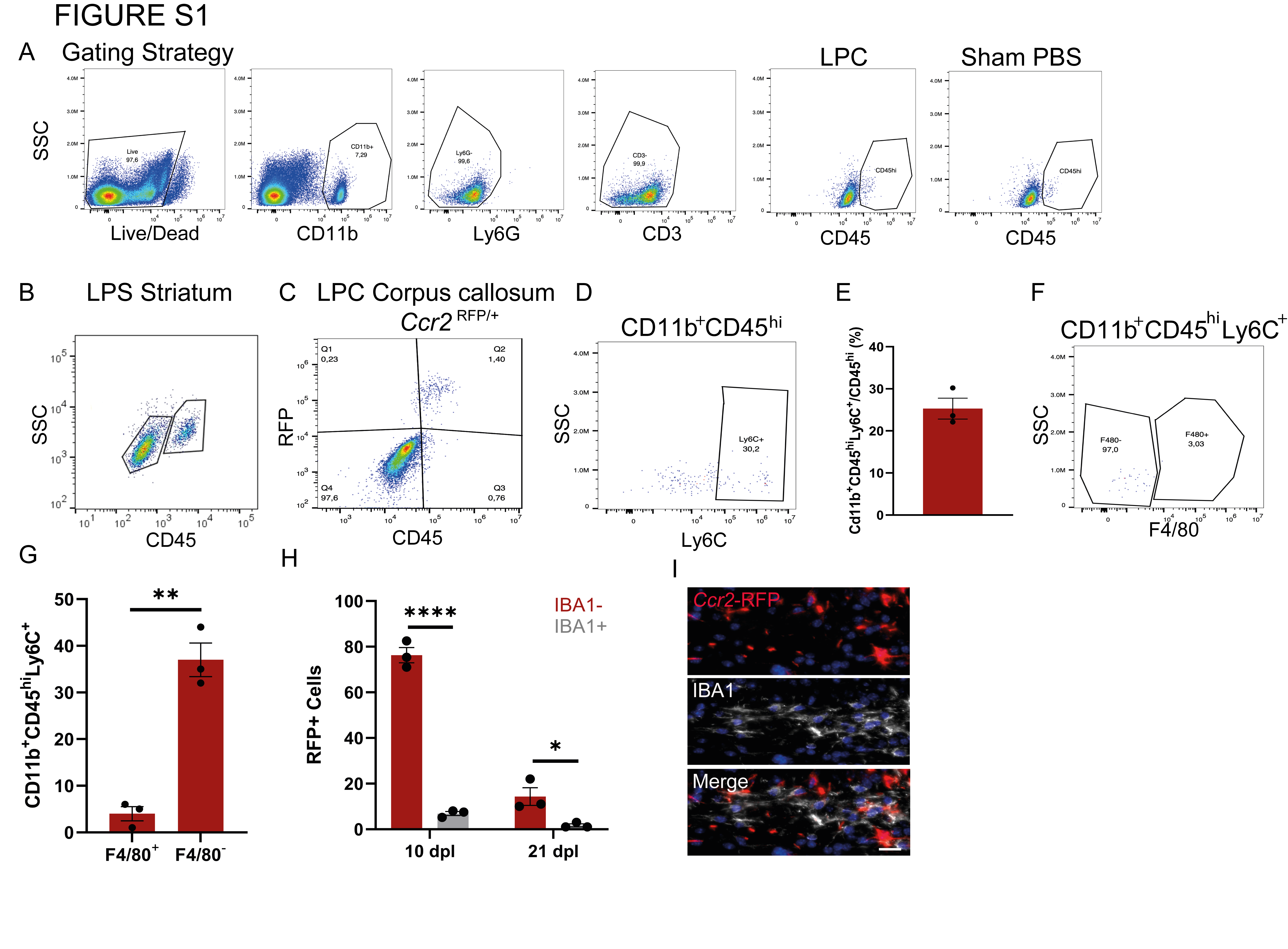

Supplement: S1 Fig — (A) Gating strategy for flow and fluorescence-activated cell sorting of myeloid cells in lysophosphatidylcholine (LPC)-induced lesions vs. sham PBS-injected corpus callosum, gating on live cells, CD11b+ cells, excluding Ly6G+ granulocytes and CD3+ T cells, and distinguishing microglia from monocytes based on logarithmic differential expression of CD45. Plots represent pooling of 2 mice per condition. SSC: side scatter. (B) Validation of gating strategy in a model in which monocytes (CD11b+ CD3− Ly6G− CD45hi) are abundant, following lipopolysaccharide injection into the striatum. (C) Validation of gating strategy using LPC-injected Ccr2RFP/+ reporter mice in which all CD45hi cells are RFP+, representing pooling of 3 mouse lesions. (D) Flow cytometry plot indicating Ly6C expression by CD11b+ CD45hi cells, representing a pooling of 2 mouse lesions. (E) Mean percentage of CD45hi cells which express Ly6C ± S.E.M. Each data point represents a pooling of 2 mice, with n = 6 mice total. (F) Flow cytometry plot indicating that the majority of CD11b+ CD45hi Ly6C+ cells in lesions are negative for the macrophage marker F4/80, representing a pooling of 2 mouse lesions. (G) Mean number of lesion CD11b+ CD45hi Ly6C+ cells which are F4/80 positive vs. negative ± S.E.M. **P = 0.0011, 2-tailed Student t test. Each data point represents a pooling of 2 mice, with n = 6 mice total. (H) Mean number of RFP+ cells which are IBA1− (red) or IBA1+ (grey) per field of lesion ± S.E.M. at 10 and 21 dpl. ****P < 0.0001 (10 dpl), 0.0177 (21 dpl), 2-way ANOVA with Sidak’s multiple comparisons tests. n = 3 mice/time point. (I) Representative fluorescent images of Ccr2RFP/+ lesions stained for the macrophage marker IBA1 (white). Scale bar, 25 µm. Mice were 8–12 weeks old. Source data may be found in S1 Data. (TIF) [file pbio.3003073.s001.tif]

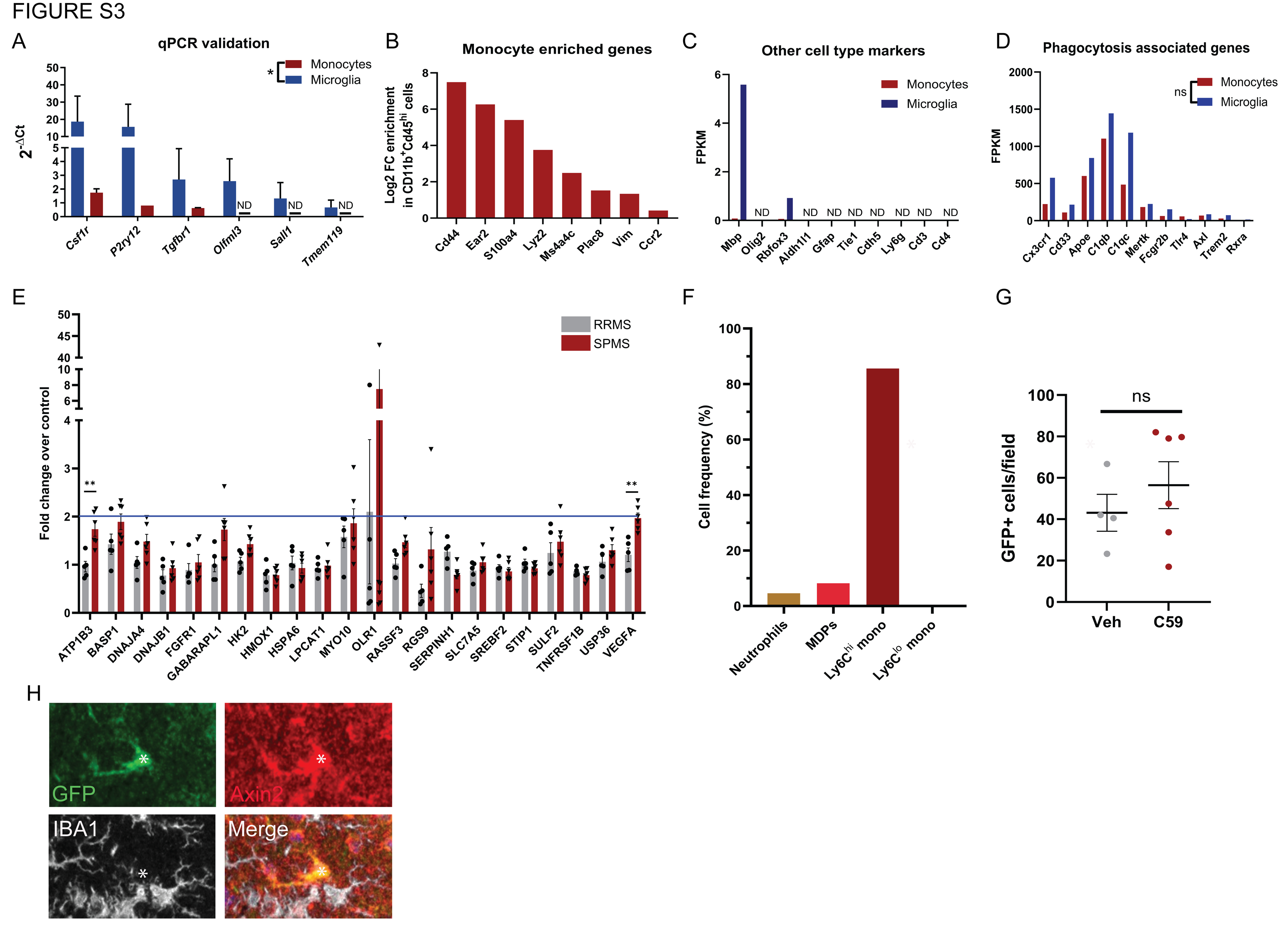

Supplement: S3 Fig — (A) Quantitative PCR of microglia signature genes ± S.E.M. in 10 dpl lesion monocytes (red) and microglia (blue). ND = not detected. *P = 0.0351; 2-tailed paired t test. n = 2–3 mice/group. (B) Log2 fold change (FC) enrichment of monocyte-enriched genes in the CD11b+ CD3- Ly6G- CD45hi vs. CD45lo cells. (C) Average FPKM of genes associated with oligodendrocytes (Mbp, Olig2), neurons (Rbfox3), astrocytes (Aldh1l1, Gfap), vascular cells (Tie1, Cdh5), granulocytes (Ly6g), and T cells (Cd3, Cd4) in monocytes (red) and microglia (blue) in lesions at 10 dpl. ND = not detected. (D) Average FPKM of phagocytosis-associated genes in monocytes (red) and microglia (blue) in lesions at 10 dpl. P = 0.0835; 2-tailed paired t test. (E) Mean expression of Wnt-associated genes in blood monocytes from individuals with relapse-remitting MS (RRMS; EDSS ≤ 2; n = 5) and secondary progressive MS (SPMS; EDSS ≥ 6; n = 6) which showed a <2-fold increase over healthy control (n = 4), represented as FC over control. Brown-Forsythe and Welch ANOVA with Dunnet’s T3 multiple comparisons test; P = 0.0325 (ATP1B3), 0.0376 (VEGFA). (F) Proportion of cells, indicated as cell frequency (%), from MACS-sorted bone marrow cells prior to transplant into Ccr2−/− mice. Neutrophils (CD45+ CD115- Ly6Ghi); MDP; monocyte dendritic cell progenitor (CD45+ CD115+ CD11B- Ly6Glo Ly6Clo); mono: monocytes (CD45+ CD115+ CD11B+ Ly6Glo, Ly6Chi vs. Ly6Clo). (G) Mean number of GFP+ cells per lesion field in Ccr2−/− mice injected with C59- or vehicle-treated monocytes. P = 0.4236; unpaired 2-tailed Student t test. n = 4–6 mice/group. (H) Transplanted monocytes (GFP+ ; asterisk) in lesions positive for Axin2 (red) but negative for IBA1 (white). Mice were 8–12 weeks old. Source data may be found in S1 Data. (TIF) [file pbio.3003073.s003.tif]
