# Supplementary material for: Monocyte-secreted Wnt reduces the efficiency of central nervous system remyelination
Source: PLoS Biol. 2025 Apr 15;23(4):e3003073. doi: 10.1371/journal.pbio.3003073 (PMC12052099; doi:10.1371/journal.pbio.3003073)
Supplement: S1 Table — Information for samples from healthy controls and people with relapse-remitting multiple sclerosis and secondary progressive multiple sclerosis: age, sex (F: female), time since last relapse, and expanded disability status scale (EDSS) score. (PDF) [file pbio.3003073.s004.pdf]

Table S1. Blood monocyte sample information

Category	Sample	Age	Sex	Time since last relapse (months)	EDSS
**Control**	1	60	F	-	-
	2	44	F	-	-
	3	35	F	-	-
	4	51	F	-	-
**RRMS**	1	41	F	3	0
	2	54	F	5	2
	3	26	F	0.5	0
	4	39	F	2	0
	5	66	F	1.5	0
**SPMS**	1	49	F	-	6.5
	2	47	F	-	6.5
	3	47	F	-	6.5
	4	47	F	-	6.5
	5	49	F	-	6
	6	56	F	-	6
