# Supplementary material for: Monocyte-secreted Wnt reduces the efficiency of central nervous system remyelination
Source: PLoS Biol. 2025 Apr 15;23(4):e3003073. doi: 10.1371/journal.pbio.3003073 (PMC12052099; doi:10.1371/journal.pbio.3003073)
Supplement: Attachment — Submitted filename: Hill Response to Reviewers R1.pdf [file pbio.3003073.s009.pdf]

We appreciate the reviewers' comments that our manuscript presents '*well-conducted and rigorous studies with several exciting implications*', experiments are '*quite elegant'*, data are '*clear, concise, and the interpretations of the results measured',* and our study would '*interest a wide readership from neuroscience and immunology-related fields*'. We appreciate the comment that our study appears suitable for the scope of PLoS Biology short reports, and we have further improved our manuscript by addressing the reviewers' comments with 8 new *in vivo* experiments, 13 new analyses, and 29 new figure panels. A summary of new data is found below, followed by a point-by-point response to individual reviewer comments. Changes in the manuscript have been highlighted in yellow to facilitate review.

1) **SUMMARY OF NEW DATA**:

- **<u>7 experiments to confirm identity of monocytes in remyelinating lesions:</u>**
  - **Gating strategy detects classical monocytes:** In addition to our original panel identifying lesion monocytes as Cd11b$^+$CD3$^-$Ly6G$^-$CD45^{hi}, we have now added in the classical monocyte marker Ly6C. This confirmed that classical monocytes are present within lesions.
  - **All cells in the CD11b$^+$CD45^{hi} gate are classical monocytes**: Using a *Ccr2*$^{RFP/+}$ reporter mouse to track the infiltration of classical monocytes into lesions, we confirm that all lesion Cd11b$^+$CD45^{hi} cells are RFP+, thus are not microglia-derived.
  - ***Ccr2*$^{-/-}$ have reduced classical monocyte recruitment to remyelinating lesions:** Using the revised flow cytometry panel above, we assessed classical monocytes in lesions of *Ccr2*$^{-/-}$ mice, which are well established to have impeded egress of monocytes from bone marrow. As expected, *Ccr2*$^{-/-}$ lesions have a significant reduction in these cells compared to wildtype lesions.
  - **Significantly lower monocytes in sham lesions:** Sham controls in which PBS was stereotaxically injected into the corpus callosum of wildtype mice, which does not cause demyelination but does cause some minimal damage from the needle, demonstrated significantly less classical monocytes than in LPC-lesioned mice. We further confirm using *Ccr2*$^{RFP/+}$ mice that no RFP+ cells are detected in contralateral non-lesioned corpus callosum. Together, these findings indicate that the cells in the LPC experiment are largely present as response to demyelination.
  - **Monocytes do not differentiate into macrophages in remyelinating lesions:** We assessed whether monocytes are differentiating into macrophages by investigating macrophage marker expression by i) flow cytometry for F4/80 in wildtype and *Ccr2*$^{-/-}$ lesions, and ii) immunofluorescence for IBA1 in *Ccr2*$^{RFP+}$ cells in lesions. Surprisingly, monocytes do not differentiate into macrophages in remyelinating lesions. We note that this was an assumption which had never been assessed in prior remyelination studies, which used pan myeloid cell markers for identification. Considering this finding, we replaced our terminology of 'monocyte-derived macrophages (MDM)' with 'monocyte' in the manuscript.
- <u>2 experiments for further assessment of monocyte recruitment to remyelinating lesions</u>:
  - **Monocytes are present over the course of remyelination:** Using lesioned *Ccr2*$^{RFP/+}$ mice, we quantified RFP+ cells in the lesion over time, when remyelination initiates, and when remyelination is complete. This indicated that monocytes are present throughout remyelination yet decrease over time as remyelination progresses.
  - **Equal recruitment of transplanted monocytes following pharmacological inhibition of Wnt release:** We quantified the injected monocytes (GFP+) recruited to the lesion site in the C59-treated (WNT release inhibition) vs vehicle-treated monocyte conditions, and found no significant difference in densities of lesion GFP+ cells.

Cont'd on next page…

- <u>**3 assessments of Wnt-responsive cells in remyelinating lesions:**</u>
    - **Monocyte Wnt signalling is detected throughout remyelination:** Quantifying protein expression of the Wnt target Axin2 in lesion monocytes (*Ccr2*$^{RFP+}$) revealed Wnt signalling in monocytes at both the initiation and completion of remyelination, with decreasing numbers as remyelination progresses.
    - **Oligodendrocyte lineage cells are Wnt-responsive during remyelination**: Oligodendrocyte lineage cells were Axin2+ during remyelination.
    - **Oligodendrocyte lineage cell Wnt signalling is decreased when lesion classical monocytes are reduced**: A reduction in Axin2+ oligodendrocyte lineage cells was observed in *Ccr2*$^{-/-}$ lesions vs wildtype control, indicating the contribution of monocytes on oligodendrocyte lineage Wnt signalling.
- <u>**5 assessments of oligodendrocyte lineage cell and myelin responses following monocyte manipulation:**</u>
    - **Monocytes influence oligodendrocyte differentiation at remyelination initiation**: In *Ccr2*$^{-/-}$ mice, mature oligodendrocytes were significantly reduced at the initiation of remyelination, then normalized to wildtype levels by remyelination completion. This indicates that monocytes promote oligodendrocyte differentiation at remyelination onset.
    - **Remyelination time course assessed by electron microscopy**: Data from our first submission indicated that at the onset of remyelination, *Ccr2*$^{-/-}$ mice have reduced myelin protein expression. We now show that despite this, the number of myelinated axons is unchanged vs lesioned wildtype mice at this time. Given the abovementioned reduction in oligodendrocytes, this indicates compensation by oligodendrocytes in making more myelin. This postulate was confirmed by analysis at remyelination completion, when we observed no change in oligodendrocytes but increased myelinated axons in *Ccr2*$^{-/-}$ mice. Together, this suggests that monocytes normally limit myelin production by oligodendrocytes during remyelination, thereby reducing its efficiency.
    - **Monocytes regulate myelin production, but not oligodendrocyte differentiation, through Wnt signaling**: Both transgenic and pharmacologic *in vivo* models of monocyte loss of function of Wnt release showed no significant change in mature oligodendrocytes in association with enhanced remyelination. This indicates that monocyte-derived Wnt suppresses remyelination independent of an effect on oligodendrocyte differentiation, therefore through impeding myelin production.
    - **Remyelination time course assessed by myelin protein expression**: Our first submission showed that myelin protein MAG is reduced in *Ccr2*$^{-/-}$ mice at the onset of remyelination. We now include data showing that this reduction is maintained by 21 dpl, yet at both time points this is dissociated from remyelination as indicated by electron microscopy. Our findings are consistent with MAG global knockouts known to have excess myelin production.
- <u>**Associating MS blood monocyte Wnt-related gene expression with disability**</u>:
    - Given that remyelination associates with better clinical presentation in MS, and remyelination efficiency decreases with MS progression, we assessed how blood monocyte Wnt signatures change with increasing disability/ progression in MS. We improved on our previous experiment by now including 11 MS samples: 5 relapse remitting MS samples (disability score under 2) and 6 secondary progressive MS samples (disability score over 6). This identified 14 Wnt-related genes with >2 fold increase over healthy controls that are significantly upregulated in progressive MS.

2) RESPONSE TO REVIEWERS:

Reviewer 1:

1.1 Despite the strong connection between the role of Wnt signaling pathway in slowing CNS MDM-regulated remyelination, the authors provide little context or discussion of downstream mechanism or effector function of Wnt signaling in slowing of remyelination, and how their results expand on previous findings. In addition, what Wnts and effector cells (or autologous signaling) do the authors think are in play? This would strengthen the Discussion section, and better contextualize the impact of their findings, and suggest future directions for this work.

We agree that the potential downstream mechanisms of monocyte Wnt activation during remyelination are interesting to discuss. To complement our data showing Wnt signaling in monocytes through Axin2 positivity, we now include data showing Axin2+ oligodendrocyte lineage cells during remyelination which are decreased when classical monocytes are reduced (**new Fig.2J-K**), together indicating both autocrine and paracrine monocytic Wnt signaling during remyelination. As suggested, we have now added to the **Discussion** the following paragraphs on potential downstream mechanisms and effector functions of WNTs in monocytes in regulating remyelination, and how this relates to the known literature.

'The downstream activation of the Wnt pathway in monocytes during remyelination suggests autocrine signalling. In other tissues, Wnt signalling in monocytes regulates gene expression to influence phagocytosis and release of both pro- and anti-inflammatory cytokines. We did not observe changes in myelin debris clearance in $Ccr2^{-/-}$ lesions following demyelination, demonstrating that the Wnt-driven impact of monocytes on remyelination is independent of this process, and pointing to the possible release of a Wnt-regulated factor restricting myelin production. In parallel, monocyte-released Wnts may directly influence other neural cells during remyelination. We observed that microglia do not express Wnt receptors nor downstream effectors during remyelination thereby ruling out their response to monocyte-derived Wnts, in contrast to the microglial response to Wnts following developmental white matter injury. However, oligodendrocyte lineage cells showed Axin2 reactivity during remyelination, which was decreased when monocyte numbers were reduced. Wnt signalling in OPCs is known to impede remyelination by reducing oligodendrocyte differentiation, although we found no significant reduction in mature oligodendrocytes when monocyte-mediated Wnt release was blocked. Rather, we saw an increase in remyelination, which indicates a new role for Wnt signalling in regulating myelin production by oligodendrocytes during remyelination."

1.2 To link Wnt signaling more firmly to MDM-mediated inhibition of remyelination, there are two potential approaches the authors can take:

a. The Csf1r-iCre;Porcfl/fl mouse knocks out WNT signaling in both MDMs or microglia. A prudent control experiment to more robustly show that WNT signaling in microglia has no effect on remyelination is a microglia-specific Porc knockout, perhaps by using the Tmem119-Cre line available through the Jackson Labs. This is very time intensive and may not be required if the authors expand their adoptive transfer approaches

b. An alternative explanation for why Wnt inhibitor pretreatment resulted in less remyelination after MDM engraftment is that there are fewer MDMs in the lesion site. Quantifying the total number of C59-treated Csf1r-eGFP+ MDMs engrafted (via quantifying total eGFP+ cells counterstained with a nuclear marker) in/around regions of high MAG or MBP staining would

better demonstrate that WNT signaling inhibition rather than lack of engrafted monocytes drives increased remyelination.

We thank the reviewer for these suggestions. To address option ‘a’, we attempted to induce recombination in the *Tmem119*-CreERT line using various routes of tamoxifen preparation (corn oil, sunflower oil), administration (subcutaneous, intraperitoneal, oral gavage), dosage frequency (3 vs 5 consecutive days), and volume (200-340 µl), including the tamoxifen regimen recommended to us by the researcher who generated the line [*Kaiser et al, 2019, eNeuro*]. None of these strategies induced Cre expression in the white matter, in contrast to the positive control Cre stain from another model [**see A, B below**]. To rule out a potential issue with obtaining the correct mice from the supplier, we confirmed the correct insertion position of the Cre by PCR (320 bp for the Cre allele, vs 400 bp for the wildtype allele) [**C**]. Our finding of poor recombination efficiency in *Tmem119*-CreERT mice is consistent with findings from others using this line and other microglia-specific Cre lines in adult mice [*Faust et al, 2023, Cell Rep; Bedolla et al, 2024, Cell Rep*]. Therefore, it was not technically possible for us to generate a microglia-specific conditional knockout.

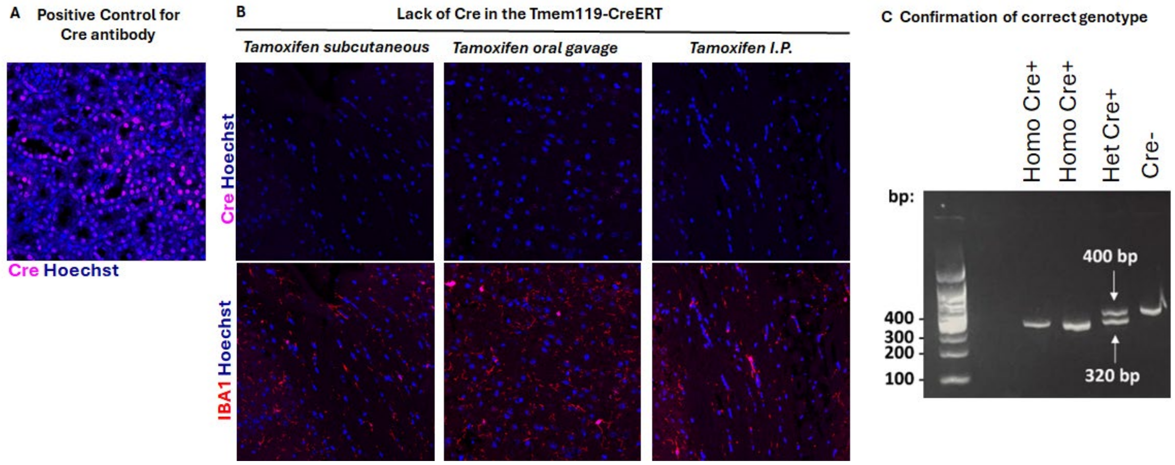

As suggested, we addressed option ‘b’ and quantified the total number of lesion GFP+ cells in the C59- vs vehicle-treated groups. Although there was a range in the number of GFP+ cells detected, this range was comparable between the two treatment groups, and there was no significant difference in recruitment of cells (**new Fig.S3F**). This indicates that the impact of C59-treated monocyte injection on remyelination was not due to fewer monocytes in the lesion.

As for a few minor comments/edits:

1.3 Typo on page 3, line 17: "With advent" should be changed to "With the advent".

The correction has been made.

1.4 Add the ages of the mice for all animal-based experiments within the Results section (or within the figure legends for each corresponding dataset) as opposed to this only being listed in the Methods section increases the rigor.

The ages of the mice have been added to the figure legends.

1.5 In the legend for Figure 1B [now 1E], describe the abbreviation in the y-axis.

SSC has been defined as side scatter in the legend.

1.6 The current design/presentation of the heatmaps in Figure 2C is confusing and difficult to interpret. Use of a less subtle gradient scale for the FC values, as well as a description in the figure legend for how to interpret said scales for both the microglial and MDM datasets would improve readability and interpretation.

As requested, we have adjusted the heatmaps and added in a description of interpretation in the figure legend. The Log2 fold change values were generated by comparing microglia transcriptomes to monocyte transcriptomes; the positive values indicate an upregulation (enrichment) in microglia, whereas negative values indicate a downregulation in microglia therefore an enrichment in monocytes. We have now chosen a rainbow gradient scale to better illustrate the range in gene expression, where red indicates highest enrichment, and purple indicates the lowest enrichment.

1.7 For the parts of a whole graphs in Figures 3A &B, add the percentile values over the "Wnt-related genes" and "Others" portions of the graphs.

We have now indicated the percentile values over the portions of the pie charts.

1.8 For the analyses discussed and presented in Figure 3C, is there a reason only the group analysis of all 5 WNT genes in MS patients v. Ctrls is showed, as opposed to presenting the results of the individual comparisons of expression levels for MS v. Ctrl for each gene? If that statistical data is available, please report it in the supplement.

In response to other reviewer suggestions, we have redone the blood monocyte analyses to increase our n numbers (**new Fig.3C**). We now include 11 MS cases, 5 with relapse-remitting MS (RRMS) (disability score EDSS 0-2) and 6 with secondary progressive MS (SPMS) (EDSS >6). This allowed us to compare gene expression between healthy control and MS, and how this changes with increasing disability (SPMS vs RRMS) when we know remyelination often fails. To identify genes upregulated in monocytes in MS, we selected a threshold of 2-fold increase in expression in progressive MS vs healthy control, which indicated 18 genes (Fig.3C). To then compare how these genes change in expression with increasing disability in MS, we performed a Brown-Forsythe and Welch ANOVA with Dunnet's T3 multiple comparisons test between RRMS and SPMS, which is best suited for >3 sets of unpaired measurements where we do not make the assumption of equal variances between groups. This indicated that 14 Wnt-related genes are significantly upregulated in blood monocytes in SPMS vs RRMS.

1.9 List the alpha values used for all statistical analyses in the Methods sections.

We had already indicated in the methods section that p values <0.01 were considered significant for the RNA sequencing bioinformatics (in the 'Bioinformatics' section), and <0.05 were considered significant for all other analyses in the paper (in the 'Statistics' section).

1.10 List catalogue/product numbers for all antibodies used across experiments and the strain numbers for all mouse lines purchased from the Jackson Lab and used in this study within the Methods section.

We have now included catalogue numbers for all antibodies and strain numbers for mouse lines in the Methods section.

1.11 Page 12, Methods, line 20: add the concentration for the PBS used here.

This has now been indicated as 1X.

1.12 Typo on page 13, Methods, line 14: change "Ultrathin" to "Ultra-thin".
This has now been corrected.

Reviewer 2:

2.1 This manuscript reported blocking monocyte egress by using the Ccr2 knockout mice leads to a pro-myelination effect at the late stage. The key message of this manuscript is partially contradictory to previous reports showing that elimination of monocytes/macrophages resulted in hindered remyelination in demyelinating animal models (Glia, 2001 35(3), 204-212; Neurobiology of Disease, 2005 18(1), 166-175). How to understand this discrepancy? Is this related to different approaches to manipulating monocytes?

We thank the reviewer for raising the important point of the different results obtained in our study compared to previous work [*Kotter et al, 2001, Glia; Kotter et al, 2005, Neurobiol Dis*], where several technical and biological differences from our study are discussed below and now summarized in the **Discussion**.

First, there are differences in the efficiency and maintenance of monocyte lesion depletion between the studies. The prior studies depleted circulating monocytes in demyelinated rats using clodronate-liposomes injected into the blood. Clodronate liposomes do not completely deplete all monocytes. In addition, the above referenced papers indicate that intra-lesional monocyte depletion is short-lived (<10 days), followed by a compensatory <u>increase</u> in the number of lesion monocytes/myeloid cells. In light of our finding that monocytes impair remyelination, the conclusion of these papers could reflect the rebound increase in monocytes in lesions which then impaired remyelination. Moreover, clodronate liposomes may also deplete lesion microglia given that the blood-brain/spinal cord barrier damage induced by the lesion induction could allow these to enter the lesion; we have previously shown that lesion microglia can be depleted by exposure to clodronate liposomes [*Miron et al, 2013, Nature Neuroscience*]. In contrast, we use the *Ccr2*$^{-/-}$ model which continuously impedes monocyte bone marrow egress, without influencing microglia directly; we have now confirmed the reduction of lesion monocytes in this model by flow cytometry for $CD11b^{+}CD3^{-}Ly6G^{-}CD45^{hi}$ and $CD11b^{+}CD45^{hi}Ly6C^{+}F4/80^{-}$ cells (**new Fig.1H,I**).

Second, there are two important biological differences between the studies which may have contributed to the different results on late-stage remyelination. The prior studies were carried out in female rats, while our study was carried out in male mice. Although sex differences in microglial responses in specific contexts have been previously described, it is unknown whether microglia or monocytes show sex differences during remyelination. In addition, the prior studies were carried out in spinal cord lesions whereas our study was carried out in brain lesions. There is a >10X increased recruitment of monocytes into spinal cord vs brain lysolecithin-induced demyelinated lesions [e.g. our study vs *Plemel et al, 2020, Science Advances*]. Furthermore, microglia transcriptomes differ in distinct CNS regions, which begs the question as to whether the different microenvironments of brain vs spinal cord might induce differential roles for monocytes in remyelination. We consider these interesting questions beyond the scope of this manuscript to pursue in future studies.

2.2 The functional switch of monocytes during remyelination is interesting. However, the authors did not explain this phenotype. For example, is the functional switch related to different degrees of Wnt tones? Different numbers of infiltrated monocytes/macrophages?

We thank the reviewer for this interesting question. Our new data (summarized in **Fig.4L**) indicates that monocytes support oligodendrocyte differentiation at onset of remyelination, yet impede late-stage remyelination by limiting myelin production; only the latter is regulated by Wnt signaling. We reached this conclusion by carrying out 5 new analyses:

[1] We assessed oligodendrocyte differentiation in *Ccr2*$^{-/-}$ mice and saw a significant decrease at onset of remyelination, but this had caught up to wildtype by remyelination completion (**new Fig.1J-K, P**).
[2] We performed electron microscopy analyses of remyelination and found that despite the decrease in oligodendrocytes at remyelination initiation in *Ccr2*$^{-/-}$, there was no change in myelinated axons (**new Fig.1N**). At remyelination completion, oligodendrocytes were unchanged between genotypes yet there were more myelinated axons in *Ccr2*$^{-/-}$ (**Fig.1Q**). Together, this indicates increased myelin production per oligodendrocyte when monocytes are reduced, suggesting that monocytes limit myelin production.
[3] We found that reducing the ability of monocytes to release Wnts either transgenically or pharmaceutically does not influence oligodendrocyte differentiation (**new Fig.4D-E,J-K**) yet enhances remyelination, indicating that monocytic Wnt acts to limit myelin production during remyelination independent of an effect on oligodendrocyte differentiation.
[4] We assessed the numbers of infiltrating monocytes in lesions using reporter mice (*Ccr2*$^{RFP/+}$) and found that monocytes are present at both remyelination initiation and completion, yet decrease in number over time (**new Fig.1B-D**); this is consistent with our model that monocytes limit myelin production, with their decrease coinciding with increased myelin production as remyelination proceeds.
[5] We assessed whether Wnt signalling in monocytes is maintained during remyelination by quantifying Axin2+ RFP+ cells, and found that these were still present at 21 dpl, yet decreased vs 10 dpl (**new Fig.2H-I**).
This decrease in monocytes and their Wnt signaling as remyelination proceeds is consistent with our new findings showing that monocytes impede myelin production via Wnt signaling.

2. 3 High-tone Wnt signaling has been demonstrated repeatedly in demyelinating lesions that can inhibit remyelination (Figure 1). How much do monocytes contribute to the level of Wnt tone in the lesions? Given the low density of monocytes/macrophages in the lesions, do the monocytes/macrophages directly regulate OPC differentiation and myelination? Or indirectly through other cell types, like microglial cells?

To address the extent to which monocytes contribute to Wnt signalling in the oligodendrocyte lineage during remyelination, we assessed expression of the Wnt target Axin2 in Olig2+ cells comparing lesions in *Ccr2*$^{-/-}$ vs wildtype mice. We detected Axin2+ Olig2+ cells in wildtype remyelinated lesions, indicating active Wnt signaling within the oligodendrocyte lineage. These were significantly reduced by almost 75% in *Ccr2*$^{-/-}$ lesions, demonstrating the important contribution of monocyte derived Wnt to oligodendrocyte lineage Wnt signaling (**new Fig.2J**).
As outlined above, we assessed the impact of monocytes on OPC differentiation using *Ccr2*$^{-/-}$ vs wildtype lesion comparisons, and found an initial decrease in mature oligodendrocytes at 10 dpl (**new Fig.1J-K**) yet no change between genotypes at 21 dpl (**new Fig.1P**). These findings indicate the requirement for monocytes to support OPC differentiation at remyelination onset, but not at late-stage remyelination. To determine whether this impact of monocytes on OPC differentiation is mediated through monocyte Wnt signalling, we assessed mature oligodendrocytes in lesions of models where monocyte Wnt release was inhibited (*Porc* conditional knockouts, and C59-treated monocyte transfer experiments). In both models, inhibition of Wnt release in monocytes had no impact on mature oligodendrocytes (**new Fig.4D-E,J-K**). As remyelination was increased in both these models, this indicates that monocyte-derived Wnts normally impede late-stage remyelination independent of an impact on oligodendrocyte differentiation. A likely hypothesis is that this occurs through limiting myelin production by oligodendrocytes. This is supported by our electron microscopy analysis now showing that despite a decrease in oligodendrocytes in *Ccr2*$^{-/-}$ at remyelination onset (10 dpl), the number of myelinated axons is unchanged (**new Fig.1N**),

suggesting more myelin made per oligodendrocyte when monocytes are reduced. The increased myelin per oligodendrocyte is then maintained at remyelination completion (21 dpl), when there is no change in oligodendrocytes between genotypes, but myelinated axons are increased (**Fig.1P,Q**). Together, these data suggest that monocytes support oligodendrocyte differentiation at remyelination onset, yet inhibit late-stage remyelination via Wnt signalling independent of oligodendrocyte differentiation.

We agree that it is interesting to consider the indirect vs direct actions of monocytes on OPCs and remyelination. With regards to the molecular mechanism we identified in this manuscript (monocyte-derived Wnts), the lack of expression of Wnt receptors and downstream targets by lesion microglia (**Fig.2F,G**) indicate they cannot be directly responsive. However, monocytes may act through microglia through a distinct molecular mechanism. This is currently challenging to functionally investigate through microglia depletion, as we recently showed myelin abnormalities as a result of microglia depletion using either a CSF1R inhibitor or a microglia-deficient mouse [*McNamara et al, 2023, Nature*]. We suggest that identifying an additional cellular or molecular mechanism through which monocytes could interact via microglia in remyelination beyond the scope of this short report, yet interesting to pursue in future studies.

2.4. Single-cell sequencing analysis revealed that the Wnt signaling was altered in the MS monocytes of the blood samples of MS, indicating Wnt changes before infiltration into CNS. An EAE model may better mirror the change of Wnt signaling in the monocytes.

We appreciate the suggestion to investigate Wnt signaling in monocytes in EAE. Please note that the Editor has indicated to us that additional experiments using the EAE model is outside the scope of this manuscript. We would like to point out that remyelination is extremely challenging to study in EAE, due to the stochastic nature of the lesions in time and space. Using immunohistochemical methods commonly used in this model for assessing gross myelin pathology, it is not possible to distinguish incomplete demyelination from remyelination. This would require electron microscopy, and if this were done only on the thoracic spinal cord (which has the highest frequency of lesions), this would require 300,000 sections at the traditional thickness of 60 nm. In addition, there is significant loss of axons in EAE which precludes extensive remyelination.

Nonetheless, we data-mined an existing database of monocyte transcriptomes during EAE [*Lewis et al, 2014*, *J Neuroimmunology*] and found that there was no expression of Wnts nor *Axin2* prior to disease onset (7 days post immunization, disease score 0) and peak disease (14 days post immunization, disease score 3). This may be due to several reasons. First, monocytes may show differential transcriptomes based on CNS region: EAE lesions primarily occur (and were sequenced) in the spinal cord, whereas the LPC model and the MS snRNAseq examined brain lesions. Second, monocytes may have a distinct transcriptome during active demyelination vs remyelination; whereas MS lesions have concurrent de- and remyelination, there is very limited remyelination in EAE, occurring only in late-stage disease [*Mei et al, 2016, eLife*]. Altogether, we consider these findings to indicate that the EAE model would not be suitable to understand how the monocyte Wnt signature observed in MS influences remyelination in the brain.

2.5 How do monocytes regulate OPC differentiation and myelination remains puzzling from the current data set, as the changes in OPC differentiation and myelination were not thoroughly examined. For example, the densities of mature oligodendrocytes and OPCs were not assessed in Figure 1 E, G. Similarly, the myelin, OL, and OPC changes were not examined in Figure 4.

As indicated in response 2.2 and 2.3, we have now assessed OPC differentiation into mature oligodendrocytes following functional inhibition of monocyte recruitment (*Ccr2*$^{-/-}$) (**new Fig.1J,K,P**) and Wnt release (**new Fig.4D,E,J,K**). We point out that changes in myelination have been assessed by myelin protein expression (**Fig.1L,M,O; Fig.4B,C,H,I**) and electron microscopy (**Fig.1.N,Q-S; Fig.S2C-E, G-I**) in the monocyte manipulation models.

Reviewer 3

3.1 The authors' assertion that CD11b+Ly6G-CD45hi cells within the lesion correspond to monocyte-derived macrophages (MDMs) is conceptually flawed. The gating strategy does not exclude monocytes nor include specific staining for macrophage markers, making it impossible to definitively identify these cells as macrophages. The MDMs gate likely encompasses a mixture of MDMs, monocytes, and microglia, as activated microglia may also upregulate CD45 expression. Additionally, the authors' claim of validating their gating strategy using Ccr2-RFP reporter mice lacks support in the LPC model, as RFP expression in CD45hi cells is only demonstrated in a different model involving LPS injection in the striatum. Furthermore, the absence of analysis in sham mice or the contralateral corpus callosum makes it challenging to assess the extent of MDM infiltration within the lesion.

To better characterize the cells in the $CD45^{hi}$ gate, we performed 5 additional *in vivo* experiments, as detailed below.

We initially employed the $CD11b^{+}$ $CD45^{lo}$ vs $CD45^{hi}$ strategy to distinguish microglia from monocytes as this approach has been well-validated in models of injured white matter and using bone marrow chimaeras [*Min et al, 2016, Mol Brain; Mizutani et al, 2012, J Immunol; Vainchtein et al, 2014, GLIA; Cusick et al, 2013, J Virol; Yamasaki et al, 2014, J Exp Med; O'Koren et al, 2016, Sci Rep*]. This has also been previously used to distinguish microglia from monocytes during remyelination [eg. *Lloyd et al, 2019, Nature Neuroscience*; *Zhang et al, 2019, PloS Biology*; *Remington et al., 2007, Am J Pathol*] and other pathological contexts [eg. *Wheeler et al., 2020, Nature*; *Rothammer et al, 2018, Nature*; *Erny et al, 2015, Nature Neuroscience*]. Although microglia are known to upregulate CD45 following injury, it is well established that this upregulation is intermediate (termed $CD45^{int}$) and can still be distinguished from monocytes ($CD45^{hi}$) by approximately 1 log of mean fluorescence intensity by flow cytometry [*Sedgwick et al, 1991, PNAS*] (Fig.1E). The only exception is in glioma [reviewed in *Silvin et al, 2023, Cell Mol Immunol*] which is not relevant to our study. We can also exclude that border-associated macrophages are included in either gate, given the absence of expression of BAM genes *Lyve1* and *Cd38* in our sequencing dataset.

To address the reviewer's comments, we expanded on our findings by:

[1] **Lesioning *Ccr2*$^{RFP/+}$ reporter mice with LPC**. We found that all $CD11b^{+}CD45^{hi}$ cells in LPC corpus callosum lesions are RFP+ (**new Fig.S1C**). This excludes the possibility of microglia contributing to the $CD45^{hi}$ gate, as they do not express *Ccr2* and accordingly are RFP negative. This is further reinforced by our RNA sequencing and qPCR validation of $CD45^{lo}$ vs $CD45^{hi}$ gate cells; whereas some microglia-enriched genes can be expressed by monocytes under specific circumstances, *Sall1* is expressed by microglia but not adult bone marrow cells nor monocytes during inflammation [*Fixsen et al, 2023, Nature Immunology*; *Salman et al., 2018, Oncotarget*; *Buttgereit et al, 2016, Nature Immunology; Li et al, 2019, Frontiers in Cellular Neuroscience*], and was detected in our microglia gate cells but not monocyte gate cells (**Fig.2A, S3A**).

[2] **Supplementing our flow cytometry panel with a marker of classical monocytes, Ly6C**. We found that $CD11b^{+}Ly6G^{-}CD3^{-}CD45^{hi}Ly6C^{+}$ cells are present in wildtype lesions (**new Fig.S1D-E**).

[3] **Assessing the maturation of the monocytes by including a macrophage marker in the flow panel, F4/80**. Surprisingly, we found that $CD11b^{+}Ly6G^{-}CD3^{-}CD45^{hi}Ly6C^{+}$ were F4/80 negative in both wildtype and *Ccr2*$^{-/-}$ lesions (**new Fig.S1F-G**). This indicates that monocytes are not maturing into macrophages in remyelinating lesions. Importantly, prior studies tracking monocyte entry into

remyelinating lesions used markers/reporter-drivers that are expressed both by monocytes and monocytes [Mac1: *Ruckh et al, 2012, Cell Stem Cell*; Cx3cr1: *Rawji et al, 2018, Acta Neuropathologica*]; as such the maturation of monocytes into macrophages in remyelinating lesions has been assumed but never assessed. In line with our results, undifferentiated monocytes have also been detected in MS lesions through single nuclei/cell RNA sequencing [*Absinta et al, 2021, Nature; Masuda et al, 2019, Nature*]. Although our finding that monocytes do not mature into macrophages is novel and interesting, it does not impact the interpretation of the other experiments in our paper, as the RNA sequencing, conditional knockout, transplant experiments and MS gene expression analysis did not rely on monocytes having matured.

[4] **Assessing the maturation of monocytes by expression of the macrophage marker IBA1 in *Ccr2*$^{RFP/+}$ reporter mouse lesions**. To further confirm that monocytes do not mature into macrophages in lesions, we assessed their expression of IBA1 – known to only be expressed by monocytes in the brain once they mature into macrophages [*Ajami et al, 2011, Nature Neuroscience*]. We found that ~80 % of RFP+ cells were negative for IBA1 (**new Fig.S1H-I**). Together with the flow cytometry data, this confirms that monocytes do not mature into macrophages in remyelinating lesions.

[5] **Assessing monocytes in sham controls**, where PBS was injected into the corpus callosum rather than LPC. Although a small number of CD11b^{+}Ly6G^{-}CD3^{-}CD45^{hi} and CD11b^{+} CD45^{hi}Ly6C^{+}F4/80^{-} cells were detected in the sham, likely reacting to the minimal damage caused by the needle, there were less cells compared to that observed following LPC-induced demyelination (**new Fig.1H,I**). This indicates that the monocytes found in LPC lesions are largely responding to demyelination.

3.2 The poor accuracy of the gating strategy utilized in this study undermines the interpretation of the RNA-seq analysis presented in Fig. 2 and Fig. S3. The authors assert that RFP is still expressed in monocyte-derived macrophages (MDMs) within the lesion site. Therefore, a more accurate sorting strategy would involve gating on genuine macrophages (by carefully excluding both Ly6C+ and Ly6C- monocytes) and sorting RFP+ and RFP- cells separately. This method would offer greater accuracy than solely relying on CD45 staining. Alternatively, the authors could have utilized Ms4a3-Cre x R26-tdTomato mice, which recombine approximately ~95% of monocytes with negligible recombination in microglia.

As addressed in point 3.1, by combining 5 complementary approaches (flow cytometry analysis of wildtype and *Ccr2*$^{-/-}$ lesions, classical monocyte reporter tracing by flow cytometry and immunofluorescence, RNA sequencing, and qPCR validation), we have convincingly shown that the CD11b^{+}CD3^{-}Ly6G^{-}CD45^{hi} cells are monocytes. Most importantly, all CD45^{hi} cells are RFP+ (**new Fig.S1C**), indicating that RFP is still expressed by monocytes within the lesion. The maintenance of RFP expression (confirmed by flow cytometry and immunofluorescence) (**new Fig.1C-D, Fig.S1C**) is consistent with our finding that classical monocytes do not mature in remyelinating lesions (**new Fig.S1E-I**), therefore they would not be expected to reduce *Ccr2* promoter activity as occurs with macrophage maturation. Although we appreciate the suggestion to supplement our work with *Ms4a3*-Cre reporter mice, we note that this line has non-specific activity in other immune populations, and also consider generation of a new reporter line outside the scope of this short report given our extensive validation detailed above.

3.3 The authors initially suggest that Ccr2-/- mice exhibit delayed remyelination at day 10, implying a role for monocyte-derived macrophages (MDMs) in promoting myelination. However, they later show that MDMs are enriched for Wnt signature genes and that inhibiting the Wnt

pathway in MDMs accelerates remyelination at day 10. I find challenging to reconcile these two findings. The authors seem to propose a biphasic response of MDMs, beneficial at the onset of remyelination but potentially detrimental at later stages. While this interpretation is intriguing, it does not appear fully supported by the data presented in this manuscript. At the present stage, it is unclear whether MDMs even persist in the lesion beyond day 10.

We now include data showing that monocytes are present throughout remyelination. Using reporter mice in which we can visualize classical monocytes (*Ccr2*$^{RFP/+}$), we detected RFP+ cells in lesions both when remyelination initiates (10 dpl) and is complete (21 dpl) (**new Fig.1B-D**). There are other instances of small numbers of immune cells having a robust impact on remyelination [e.g. T regs, *Dombrowski et al, 2017, Nature Neuroscience*; pericytes, *Guzman de la Fuente et al, 2017, Cell Rep*]. We put forward that our proposal of a biphasic response of monocytes is supported by our new data, where monocytes promote oligodendrocyte differentiation at remyelination onset in a Wnt-independent manner, then limit myelin production during remyelination in a Wnt-dependent manner (summarized in **Fig. 4L**), supported by the following experiments:

[1] We assessed oligodendrocyte differentiation in *Ccr2*$^{-/-}$ mice and saw a significant decrease at onset of remyelination, but this had caught up to wildtype by remyelination completion (**new Fig.1J-K, P**).

[2] We performed electron microscopy analyses of remyelination and found that despite the decrease in oligodendrocytes at remyelination initiation in *Ccr2*$^{-/-}$, there was no change in myelinated axons (**new Fig.1N**). At remyelination completion, oligodendrocytes were unchanged between genotypes yet there were more myelinated axons in *Ccr2*$^{-/-}$ (**Fig.1Q**). Together, this indicates increased myelin production per oligodendrocyte when monocytes are reduced, suggesting that monocytes limit myelin production.

[3] We found that reducing the ability of monocytes to release Wnts either transgenically or pharmaceutically does not influence oligodendrocyte differentiation (**new Fig.4D-E,J-K**) yet enhances remyelination, indicating that monocytic Wnt acts to limit myelin production during remyelination independent of an effect on oligodendrocyte differentiation.

Altogether, this data indicate that monocytes have an initial beneficial impact on oligodendrocyte differentiation, yet this is not mediated by their Wnt release. Monocytes then ultimately impede myelin production via Wnt signaling.

This data provide a molecular mechanism (Wnt signalling) for the deleterious impact of monocytes on myelin production during remyelination, but as the reviewer points out, does not explain the beneficial effect of monocytes on oligodendrocyte differentiation at remyelination initiation. A prior study suggested that monocytes may regulate remyelination via the growth factors IGF1 and TGFB1 [*Kotter et al, 2005, Neurobiol Dis*], although monocyte-derived IGF1 or TGFB1 were not functionally tested for their influence on remyelination. Nonetheless, these molecules are known to promote remyelination initiation [*Mason et al, 2003, J Neurosci*; *Hamaguchi et al., 2019, eLife*] and we find they are expressed in monocytes at remyelination initiation in our dataset (10 dpl) [FPKM 58.22 and 230.42 respectively] at a much higher level than microglia [Log2FC 3.82 and 5.615, both $P<0.05$]. We now put forward in the **Discussion** this possibility. We consider this an interesting future direction to pursue.

3.4 Lastly, it is concerning that many critical experiments described here show statistics on n=3 mice/group, indicating that the experiments were performed only once. This raises questions about the reliability and reproducibility of the results. Increasing the sample size or replicating the experiments would improve the robustness of these findings.

We highlight that we revised our flow cytometry experiments to have 2 pooled mouse lesions, repeated 3 times, in order to increase the ability to quantify monocytes. To address the reviewer's concern about the robustness of observations in experiments with n=3 mice/group, we performed power calculations on these datasets by the normal approximation method using a two-sided confidence interval of 95%. Of the experiments showing statistical significance, these had a statistical power of 100% (Fig.1C), 98% (Fig.1K), 99.9% (Fig.1Q), 100% (Fig.2I), 97.9% (Fig.2J), and 86% (Fig.4C), well above the recommended 80% power for confidence in statistical analyses. For the remaining experiments which indicated no statistical difference, we used the open-source calculator SS Mean to determine the optimal sample size that would be required to reach statistical significance at p<0.05, at a power of 80%. This was calculated to be 306 mice (Fig.1N), 548 mice (Fig.1R), and 68 mice (Fig.4). We would consider it biologically insignificant to require this large n number to see a significant difference, in addition to the lack of feasibility of these experiments. All other experiments included a larger sample size. We followed the guidelines of the UK NC3Rs to reduce the number of animals used in experimentation as much as possible, and are therefore satisfied with having done so whilst being statistically powered to detect statistical differences in our experimental groups using a reasonable n number.

Minor comments:
3.5 Why do the authors sometimes use the one-tailed T test instead of two-tailed? How can the authors assess the normal distribution when the N size is 3?
We used a 2-tailed t-test when comparing mean values across 2 groups, whereas we used a 1-tailed t-test when performing statistical tests on values normalized to a control group (e.g. value of 1 when fold over control). The Shapiro-Wilk test can perform normality tests on sample sizes of 3.

3.6 Authors stated that circulating monocytes are normally absent in the Ccr2-/- mice (page 8). Monocytes are not "absent" in these mice. They are just reduced.
We have corrected the statement to indicate that classical monocytes are reduced in the circulation and within lesions in the knockout mice, throughout the manuscript.

3.7 In Fig. S3F, the authors gated DCs as CD45+CD115+Ly6Ghi cells. These are neutrophils. DCs do not express Ly6G.
We apologize for the typo and have corrected this.

3.8 Panel Fig. 2I displays fluorescence images of RFP+ macrophages within the lesion, but these images do not appear convincing. I can barely distinguish the shape of the cells. The low RFP signal is probably due the antigen retrieval step. I am wondering if employing Ccr2-GFP or Ms4a3-Cre : tdTomato mice could enhance the quality of these images.
We have replaced the images and recommend viewing the high resolution version of the figures.

Reviewer 4

4.1 Figure.1 There is very little characterisation of the macrophage response to LPC. Firstly, Fig1B shows almost no macrophages (MDM) recruited to the lesion, and at least one mouse has zero cells recruited. The representative FACS plot shows what one would expect from a naïve mouse, as there are always a few CD11b+ CD45hi cells in these preps. In an area that has been denuded of its myelin, you would expect MDM recruitment, but it certainly is not convincing here. The authors must show more evidence that MDMs are actually recruited to the site and that in the ccr2 -/- mice, the cells are no longer recruited. This is an absolutely crucial bit of data to verify that the downstream effects on myelin are due to absence of cells in the knockout. There are many ways to distinguish microglia and MDMs now, and the ccr2 +/rfp is one of them (as they show in Fig.2 G). Without these data, the idea that recruited monocytes at the lesion are responsible for remyelination kinetics is difficult to support.

We thank the reviewer for these suggestions, which we have addressed with 6 new *in vivo* experiments outlined below. Together, our findings indicate that monocytes are present in lesions throughout remyelination, primarily infiltrating in response to demyelination. We acknowledge that the numbers of monocytes are low, although we point out that no mouse had 0 lesion monocytes.

[1] To overcome the issue of visualizing low numbers of monocytes by flow cytometry, we **repeated lesioning experiments for flow cytometry whilst pooling 2 mice per group**, and repeating the experiment 3 times each for the wildtype LPC lesion group, the *Ccr2*$^{-/-}$ LPC lesion group, and the newly added sham PBS-injected group (**new Fig.1E-I; new Fig.S1D-G**).

[2] We confirmed that classical monocytes are recruited to remyelinating lesions using a complementary approach suggested by the reviewer, by **quantifying the number of *Ccr2*$^{RFP+}$ cells in lesion tissue by immunofluorescence (new Fig.1C-D)** and detected positive cells in lesions throughout remyelination.

[3] By pooling 3 ***Ccr2*$^{RFP/+}$ lesions for flow cytometry**, we detected a robust number of RFP+ cells within lesions, which were all CD11b$^+$CD45^{hi} (**new Fig.S1C**).

[4] As requested, we confirmed **reduced classical monocyte lesion recruitment in *Ccr2*$^{-/-}$ lesions**. We increased confidence in our flow cytometry panel by detecting the classical monocyte marker Ly6C (**new Fig.S1D-E**) and the absence of expression of the macrophage marker F4/80 (**new Fig.S1F-G**). Compared to wildtype lesions, we confirmed that *Ccr2*$^{-/-}$ lesions have reduced CD11b$^+$CD3$^-$Ly6G$^-$CD45^{hi} cells, and of those, reduced Ly6C$^+$F4/80$^-$ cells (**new Fig.1H,I**). This confirms that the knockout reduces classical monocyte recruitment to lesions, as expected based on previous studies [*Saederup et al 2010, PLoS One; Ruckh et al, 2012, Cell Stem Cell; Lampron et al, 2015, J Exp Med*].

[5] We assessed **sham PBS-injected corpus callosum** for monocytes, which causes some minimal tissue damage from the needle. Using flow cytometry to compare to LPC-induced lesions, sham tissue had less monocytes (Cd11b$^+$CD3$^-$Ly6G$^-$Cd45^{hi} cells, and of those, less Ly6C$^+$F4/80$^-$ cells; **new Fig.1H,I**).

[6] We also show by immunofluorescence that there are **no RFP+ cells in contralateral non-lesioned white matter** (with underlying choroid plexus providing a positive control), indicating that monocytes are not normally recruited to uninjured tissue (**new Fig.1D**).

We point out that the low number of monocytes in our focal brain LPC lesions (500 µm x 500 µm) is consistent with those observed in demyelinated lesions of the corpus callosum in the cuprizone model (average 500 total Cd11b$^+$Cd45^{hi} cells from the <u>entire</u> corpus callosum at onset of remyelination [*Remington et al, 2007, Am J Pathol; Lampron et al, 2015, J Exp Med*]). Conversely, spinal cord LPC

lesions show approximately 10X more monocyte recruitment vs brain LPC lesions [our study vs *Plemel et al, 2020, Science Advances*], consistent with robust monocyte recruitment in spinal cord in traumatic injury and EAE. This indicates an interesting difference in CNS region-specific recruitment of monocytes for future investigation. Nonetheless, our 3 loss of function models included in our manuscript indicate the functional importance of monocytes in brain lesions, despite their low numbers. This complements the known beneficial effect of T regs in remyelination despite the very low density of 1 cell/mg of lesion tissue [*Dombrowski et al, 2017, Nature Neuroscience*].

4.2 If it can be robustly shown that monocytes are recruited, it still does not automatically follow this MDMs affect remyelination. In E to H, how do we not know that remyelination is not just due to lack of appropriate clearance responses in the acute phase after LPC injection? i.e. MDMs will be recruited to the lesion as early as two days after demyelination in WT mice. In Ccr2 $^{-/-}$ they should not be. Therefore, these results are just as likely to be due to inappropriate clearance of debris in the acute phase. With the Ccr2$^{-/-}$ strategy, the authors cannot decouple inadequate clearance from regenerative response after clearance.

We thank the reviewer for this interesting question as to whether monocytes influence remyelination via debris clearance or another mechanism. Our original submission included images of myelin protein staining at the peak of demyelination (3 dpl), which indicates no accumulation of myelin debris in the *Ccr2*$^{-/-}$ vs wildtype lesions (**Fig.S2A**). We now supplement this with new data using a complementary method commonly used to assess myelin debris, Oil Red O staining, and also observe no overt accumulation of debris and no difference between genotypes at 3 dpl (**new Fig.S2B**). Altogether, these findings indicate that the impact on remyelination in *Ccr2*$^{-/-}$ mice is not due to inadequate clearance of myelin debris in the acute phase. Furthermore, we include new data in the *Ccr2*$^{-/-}$ mice on oligodendrocyte differentiation (**new Fig.1J-K,P**) and myelin production at 10 and 21 dpl (**new Fig.1N, 1Q-S**), indicating impacts on regenerative responses that are known to occur after myelin debris clearance.

4.3 Can the authors show that in the days 1-10 that loss of monocyte recruitment does not affect demyelination?

As indicated above, we included in our original submission images of myelin protein at the time of peak demyelination, 3 dpl, which indicated a reduction of myelin basic protein in the corpus callosum in both genotypes (**Fig.S2A**) pointing to no differential impact on demyelination in *Ccr2*$^{-/-}$ mice. Furthermore, we now include semi-thin sections at 10 dpl which indicate lesioning in both genotypes, evidenced by cellular accumulation and tissue disruption visualized by toluidine blue staining (**new Fig.S2F**). We did not expect to see impaired demyelination in this model, as LPC works as a detergent to dissolve myelin and this demyelination is not considered to be dependent on monocytes (e.g. it is effective in *ex vivo* brain explants, where monocytes are absent).

4.4 For the authors to say the 'tempo' of remyelination is changed would need them to use the same measurement across time. At the moment, MAG staining is at 10dpi as an 'early remyelination marker', but how well does this correlate with myelinated axons? If they authors want to make a firm statement about remyelination 'tempo', the authors need to do a time course with between day 10 and 21 with the same readout.

We note that we have replaced the term ‘tempo’ with ‘efficiency’ throughout our manuscript. Nonetheless, as requested we have the same analyses at 10 and 21 dpl, for oligodendrocyte maturation and myelin assessment by electron microscopy analysis and MAG staining, as outlined below.

[1] **Oligodendrocyte maturation**: We now document a relative decrease in mature oligodendrocytes in *Ccr2*$^{-/-}$ vs wildtype lesions (CC1+Olig2+) at 10 dpl (**new Fig.1J-K**). By 21 dpl, this has normalized and there is no difference between genotypes (**new Fig.1P**). This indicates a requirement for monocytes for oligodendrocyte maturation at the onset of remyelination, but not for late-stage remyelination.
[2] **Electron microscopy**: We performed new electron microscopy analysis of remyelination at 10 dpl in *Ccr2*$^{-/-}$ vs wildtype control, to complement the analysis performed at 21 dpl in our first submission. As expected, the number of myelinated axons increased from 10 to 21 dpl in wildtype control, as remyelination proceeds (**new Fig.1N vs Fig.1Q**). However, despite the decrease in oligodendrocytes in *Ccr2*$^{-/-}$ lesions at 10 dpl, the number of myelinated axons was unchanged (**new Fig.1N**). We confirmed the presence of lesions in 10 dpl resin blocks (the same ones used for electron microscopy analysis) in both genotypes by imaging semi-thin (1 µm) toluidine blue stained sections (**new Fig.S2F**). This indicates that oligodendrocytes compensated for their decrease by making more myelin, and suggests that monocytes normally repress myelin production. In support of this postulate, the number of oligodendrocytes are unchanged at 21 dpl, yet more myelinated axons are observed in *Ccr2*$^{-/-}$ lesions (**Fig.1Q,S**).
[3] **MAG expression**: We performed new MAG staining at 21 dpl, to complement our prior observation of reduced MAG at 10 dpl in *Ccr2*$^{-/-}$ lesions. We observed a maintained reduction of MAG in *Ccr2*$^{-/-}$ vs wildtype lesions at 21 dpl (**new Fig.1O**). Therefore, there is a dissociation between decreased myelin protein expression and increased remyelination. Our findings in *Ccr2*$^{-/-}$ lesions of reduced lesion MAG levels with increased myelin production is consistent with previous studies showing that MAG knockouts make excess myelin [*Li et al,, 1994, Nature; Montag et al, 1994, Neuron*].

4.5 Mechanism - MDMs have a WNT signature during remyelination. A large proportion of the paper depends in bulk RNA sequencing from ultra-low RNA from sorted MDMs and microglia. Much more information is needed in order to assess the validity of these experiments as they are very prone to bias in RNA amplification steps from ultra-low material. Firstly, what were the cell yields for the sorted MDM and sorted microglia?
The average cell yield for sorted microglia was 5777 per mouse, and the average cell yield of sorted monocytes was 38 per mouse. Please refer to our answer to point 4.6 below as to the reason for the unequal number of sorted microglia vs monocytes. We sequenced an average of 4 ng of RNA for microglia and 1.6 ng of RNA from monocytes. Ultra low input RNA seq is optimized to sequence samples with as little as 0.5 ng. This was sufficient for our purposes as we detected high expression of myeloid cell-associated genes in both microglia and monocyte populations (e.g. *Cx3cr1*, *Mertk*; **Fig.S3C**), and differential expression of genes known to be enriched in microglia vs monocytes (e.g. *Sall1*, *Tmem119*; **Fig.2A**).

4.6 Were mice pooled? If the data in Fig. 1C are representative, more than half the mice will have less then 50 cells (some have zero). Just a few stray nuclei of neurons/astrocytes/endothelial cells and this will massively change the gene expression profiles in the sample. Were numbers of sorted microglia equal to MDMs? If many microglia were sorted, this would reduce the noise in this group, whereas MDM group would be very much influenced by non-MDM cells.

For sequencing, the mice were not pooled to allow statistical comparisons. Although the number of lesion monocytes per mouse are low, none were 0. As indicated in response 4.1, our finding of low numbers of monocytes in demyelinated corpus callosum is consistent with prior studies, and with our new analyses of lesion monocyte densities by immunofluorescence of *Ccr2*$^{RFP+}$ cells (**new Fig.1B-D**). By flow cytometry, we now also show that all the CD11b^{+}CD45^{hi} cells that were sorted are *Ccr2*$^{RFP+}$ (**new Fig.S1C**). As neurons, astrocytes, and endothelial cells are negative for *Ccr2*, CD11b and CD45, these cells would not have been included in the sorted cells for RNA sequencing.
Nonetheless, in our original submission, we addressed the possibility of other contaminating cells in our samples by assessing expression of cell type-enriched genes: *Mbp* and *Olig2* (oligodendrocyte lineage cells), *Rbfox3* (neurons), *Aldh1l1* and *Gfap* (astrocytes), *Ly6g* (granulocytes), *Cd3* and *Cd4* (T cells). There were no transcripts of other cell types detected in the sorted monocytes samples (**new Fig.S3B**). A low level of transcript for *Rbfox3* and *Mbp* were detected in the sorted microglia samples only, which may reflect phagocytosis of debris and/or dying cells, as this has been previously published in microglia sequencing in an injury context [e.g. *Lewis et al, 2014, J Neuroimmunol*]. We have now updated this graph to include endothelial cell genes (*Tie1* and *Cdh5*) which were not detected, further confirming specificity of our sorting (**new Fig.S3B**). Importantly, we validated the Wnt signature from RNA sequencing in monocytes through Axin2 protein expression detected in in *Ccr2*-RFP+ cells in lesions (**new Fig.2H,I**), and its functional relevance in these cells using 2 *in vivo* models of loss of function of Wnt release by monocytes (**Fig.4**).
The numbers of cells sorted for microglia and monocytes were not equal, given the unequal numbers of cells found in the lesions. As microglia are >100 fold more abundant than monocytes in lesions, this would require the use of >300 mice to sort equal numbers of monocytes, which we would consider technically infeasible. We acknowledge the difficulty in assessing monocytes by flow cytometry in individual mice given the low numbers. For this reason, we repeated the flow cytometry analysis by pooling 2 mice per group (for each condition: wildtype sham PBS-injected, wildtype LPC lesions, *Ccr2*$^{-/-}$ LPC lesions) and setting a higher threshold for event detection (**new Fig.1H,I**).

4.7 There appears to be no biological, or even technical replicates. Was this done? Is it just one pooled sample? There is no report of n numbers. What are the paired t-tests done on? Using each gene as an n number? This clearly isn't correct if so.
We apologize for the lack of clarity, as we used biological replicates for the RNA sequencing and this is now indicated in the figure legends. We chose to perform a paired t-test for a grouped analysis of the subset of genes being analysed, as this is used to consider the regulation of genes as a set, rather than focus on individual genes with repeated individual tests. We chose this approach as we are interested in the Wnt pathway and not specific Wnt genes. This approach has been previously used to compare expression differences between conditions for a select group of genes following RNA sequencing [eg. *Hasel et al, 2018, Nature Communications; Qiu et al, 2018, Nature Protocols; Molina-Gonzalez et al, 2023, Nature Communications; Lloyd et al, 2019, Nature Neuroscience*].

4.8 The idea that Wnt is involved depends on the quality of the above data. Yet, the quantitative expression of Wnt is extremely low, in over half of the transcripts the authors suggest are significantly 'up regulated' are in fact, in their own words, negligibly expressed (FPKM<0.05) Fig S3D. Then, surprisingly, they appear to compare this negligible expression to that of microglia, which is zero, and therefore it is misleading to show a major fold upregulation, from microglia. Due to the extremely, extremely low expression of Wnt, and particularly if there is only one

sample, contamination from neurons and astrocytes which also express Wnts is a major concern.

As stated above, our sequencing data represented biological replicates and was not one pooled sample. We would like to point out that out of the 13 Wnts detected in lesion monocytes, 11 showed an FPKM of over 0.05 (**Fig.2F**), a commonly used threshold for gene expression from RNA sequencing analyses. The reviewer is correct in that microglia do not express most Wnts or their receptors (**Fig.2F,G**), therefore we removed the Log2 fold change comparison of expression of monocytes vs microglia and moved the raw monocyte FPKM data from supplemental material to the main figure (**Fig.2F,G**). As explained in point 4.6, there is no evidence from our RNA sequencing data to support that we have contamination of neurons, astrocytes, or other neural or immune cell types in the monocyte population sequenced. In addition, both loss of function *in vivo* models of impaired Wnt release (**Fig.4**) would not have influenced neurons or astrocytes directly, given the specificity of the transgenic and the transplant of treated monocytes. Lastly, we validated the Wnt activity in monocytes by assessing expression of a major Wnt target, Axin2, in *Ccr2*$^{RFP+}$ monocytes in lesions (**new Fig.2H-I**).

4.9 The authors attempt to validate some the sequencing by showing Axin2 protein expression in lesion MDMs using Ccr2RFP/+ reporter mice and found that '85% of the RFP+ MDMs were positive (Fig.2H-I).' What time point is this at? And crucially, is axin2 protein found in microglia, or indeed other cells?

Based on another reviewer's suggestion, we now include data to assess monocyte (*Ccr2*-RFP+) expression of Axin2 in lesions at 2 key time points in remyelination, the initiation (10 dpl) and the completion (21 dpl) (**new Fig.2H,I**). As we also now show that RFP+ cells decrease as remyelination proceeds (**new Fig.1C**), we have represented the RFP+Axin2+ data as numbers of cells per field rather than percentage for accuracy (**new Fig. 2I**). The average number of RFP+Axin2+ cells per lesion field is 42 at 10 dpl, and 8 at 21 dpl. As included in the original submission, analysis of microglia full transcriptomes show that they do not express *Axin2* (Fig.2G). However, we assessed Axin2 expression in oligodendrocyte lineage cells (Olig2+) and found ~40% of Olig2+ cells express Axin2 during remyelination (**new Fig.2J,K**). We queried whether this responsiveness was dependent on classical monocytes by staining *Ccr2*$^{-/-}$ lesions, and found a significant reduction down to 10% double positive cells detected (**new Fig.2J,K**).

4.10 The authors now turn to human data and published sequencing data sets to examine Wnt related genes in the cells in the rim of the lesion. Some more data on the two 'classical monocyte clusters (renamed 1 and 2)' would help the reader assess the data.

We have now included additional information on this dataset in the **Methods** in a new section titled 'Data-mining published monocyte transcriptomes', as follows:

'Sequencing datasets were obtained from a published study of single nuclei RNA sequencing of multiple sclerosis (MS) chronic active (i.e. 'mixed') lesions [Absinta et al, 2021, Nature]. In their study, the authors subclustered immune cells (CSF1R+, C3+, CD74+, RUNX1+) and lymphocytes (PTPRC+, CD2+, SKAP1+), then further subclustered these cells. Two clusters were annotated as monocytes based on upregulation of CD83 and lower expression (negative Z scores) of microglia-enriched genes SALL1 and TMEM119 compared to all immune cells including microglia. Peripheral immune cell signatures were further mapped onto a multimodal peripheral blood mononuclear cell (PBMC) reference dataset to confirm identities; the majority of the monocytes expressed CD14 rather than CD16, therefore representing classical monocytes.'

4.11 The authors say these data 'revealed that ~50% of genes are known to either be regulated by Wnts or regulate Wnt signalling (Fig.3A,B).' What does this mean? 50% of all genes that were measured? 50% of genes that are in the Wnt pathway are regulated? What do they mean by regulated? They are expressed as fold change, but fold change from what? Are these significantly changed.

We apologize for not clearly explaining the Wnt pathway enrichment in MS monocyte clusters. The genes included in the dataset were listed by *Absinta et al* to be in the top 100 significantly differentially expressed genes (adjusted p value <0.05) for each immune cell cluster, compared to the rest of the immune cell clusters; this represents the fold change vs other immune cell clusters. This is a standard statistical approach used to identify genes enriched in cell clusters from single nuclei or single cell RNA sequencing. In our study, we investigated the genes significantly enriched in the 2 monocyte clusters compared to other immune cell clusters. Of these genes, 50% are either influenced in function or gene expression by activation of the Wnt pathway, or in turn influence the activation of the Wnt pathway.

4.12 The histograms show the majority of these genes are close to Log2FC of 1, and non above 1.5. Convention suggests that Log2FC of 2 or 0.5 is necessary (with appropriate statistics including FDR etc.) to suggest a biological meaning. There is potentially none here.

The qPCR of monocytes referred to by the reviewer represented 4 relapse-remitting (RRMS) MS cases in our original submission. To increase confidence in our assessment of Wnt-related genes in MS blood monocytes, we repeated the gene expression analysis with 11 MS cases, including both RRMS and secondary progressive MS (SPMS), in addition to age and sex matched controls (**new Fig.3C**). This new cohort gave us more statistical power and allowed us to assess how Wnt-related gene expression changes in MS monocytes with increasing clinical disability/disease progression, as remyelination efficiency decreases with worsening disability in progressive MS [*Bodini et al, 2016, Ann Neurol*].

Samples from individuals with RRMS had low disability score (EDSS of ≤2) and those with SPMS had high disability scores (EDSS ≥6). SPMS monocytes showed increased expression of 18 genes vs healthy control at a >2-fold expression difference (**new Fig.3C**). Of these genes, 14 showed significantly increased expression in SPMS monocytes vs RRMS monocytes by Brown-Forsythe and Welch ANOVA with Dunnet's T3 multiple comparisons test. Half of these genes show an impressive upregulation of >5 fold, e.g. one gene is >1000 fold upregulated in SPMS monocytes. Together, this data indicates a significant increase in Wnt-related gene expression in monocytes with increasing MS disability/progression, when remyelination often fails. These results are consistent with our mouse experiments indicating that Wnt activity in monocytes impairs remyelination (**Fig.4**). The relatively lower gene expression levels in RRMS, when remyelination can still occur efficiently, explains why our original cohort of only RRMS patients showed relatively small changes in gene expression.

4.13 The authors also look at human blood monocytes from MS patients and five Wnt-associated genes were measured, to show increases from healthy controls (Fig.3C) but how can a Paired t-test between groups done on these data? It should be a two-way ANOVA with post hoc tests.

The graph referred to by the reviewer was removed from the revised paper. As indicated in 4.12, we have now redone our experiment and analyses on MS monocytes with a larger cohort. We performed statistical comparisons between SPMS and RRMS monocytes by Brown-Forsythe and Welch ANOVA with Dunn's T3 Multiple comparisons test, which is best suited for >3 sets of unpaired measurements where we do not make the assumption of equal variances between groups.

4.14 The final figure probes the functional consequence inhibiting Wnt activity in macrophages. Interestingly, they find that the floxed controls also change the 'tempo' of remyelination. As it 'occurred at a slower pace than expected, with poor remyelination at 14 dpl likely resulting from the FVB background'. The subsequent experiments then assess the tempo of remyelination by inhibiting Wnt signalling in MDMs. Some of these experiments are quite elegant, but there is pretty much no data on their characterization, again making them difficult to assess. How many treated cells were injected to each mouse? How consistent was their recruitment to the CNS?

We note that it is known that different mouse strains show differences in white matter development [e.g. *Goudriaan et al., 2020, Cells*; *Dean et al, 2011, Dev Neurosci*], and susceptibility to demyelination [e.g. *Yu et al, 2017, Cell & Bioscience; Skripuletz et al, 2008, Am J Pathol*]. Therefore, it is perhaps not surprising that we see differences remyelination when comparing FVB mice to C67Bl6/J mice (the latter used for all other experiments, and for the majority of remyelination studies in the literature). Nonetheless, using the *Porc*$^{fl.fl}$ mice as a control for the *Csf1r*-iCre;*Porc*$^{fl.fl}$ mice allows us to control for strain-specific differences in how remyelination proceeds.

With regards to the transplant of C59-treated monocytes injected into lesioned *Ccr2*$^{-/-}$ mice, we had provided this information in the **Methods**: '*Each mouse received 1 injection containing 500,000 cells in 100 µl of PBS*'. For clarity, we have now mentioned this in the **Results** section as well. We also include new data quantifying the recruitment of the injected cells to the CNS, and although we see a variable recruitment between mice, the range of recruited cells is comparable and not significantly different between treatment conditions (**new Fig.S3F**).

4.15 As this is critical to the claims, can the authors also show the same effect by injecting Porc fl/fl macrophages?

We appreciate the suggestion to perform transplant of *Csf1r*-Cre;*Porc*$^{fl.fl}$ monocytes into lesioned *Ccr2*$^{-/-}$ mice, to mirror the experiment we performed with reporter-labelled monocytes (*Csf1r*-eGFP) treated *ex vivo* with the Wnt inhibitor C59 prior to transplant (**Fig.4**). However, there are major technical difficulties with this suggestion such that it would require >1 year to set up and is unlikely to work, detailed below. We shared these difficulties with the editor who agreed and noted that this experiment would not be required for acceptance of the paper.

[1] The *Csf1r*-Cre;*Porc*$^{fl.fl}$ mice and *Ccr2*$^{-/-}$ mice are of different strains (FVB and C57Bl6, respectively), therefore the injected cells would likely be detected by the host immune system and destroyed. In our C59 experiment, both the donor and host mice are of the same strain, thus avoiding this problem.

[2] We would not have a robust way of tracking the conditional knockout cells to ensure that they reach the brain lesion. Whereas we had isolated cells from a reporter mouse to allow long-term GFP expression and tracking, the conditional knockout does not have an inherent reporter. Creating a reporter conditional knockout would require a triple transgenic (Cg-Tg(CAG-eGFP);*Csf1r*-Cre;*Porc*$^{fl.fl}$) which would take at least a year to generate and age up to adulthood, even prior to experimentation and analysis. Alternatives for cell tracking such as loading with dyes or latex beads would be diluted by monocyte proliferation, and are not 100% efficient thereby precluding accurate quantification of cells migrated into the lesion. In addition, these labelling approaches are often short-term in nature, and would require phagocytosis of these agents which may change the function of the monocytes prior to CNS entry.

4.16 Lastly, its extremely difficult to assess the tempo of something when only one time point is assessed, especially as the initially experiments show that in one phase it is delayed then later accelerated.

We agree with the review that comparing time points for a readout is important in assessing changes throughout remyelination. We have now performed 5 additional experiments to provide a time course of the following:

[1] Monocyte recruitment to lesions: using *Ccr2*$^{RFP/+}$ mice, we assessed monocyte recruitment to lesions over time, comparing the time point when remyelination is initiating (10 dpl) and complete (21 dpl). We observed that monocytes are present at all time points, yet decrease over time (**new Fig.1B-D**).

[2] Remyelination in *Ccr2*$^{-/-}$ vs wildtype lesions by electron microscopy: we compared the density of myelinated axons in lesions at 10 dpl and 21 dpl. As remyelination proceeds in wildtype mice, the number of myelinated axons increased. When comparing *Ccr2*$^{-/-}$ to wildtype mouse lesions, there was no difference in myelinated axon density at 10 dpl (**new Fig.1N**), whereas this was increased over wildtype at 21 dpl (**Fig.1Q**). This indicates that monocytes don't normally impede remyelination onset, but do limit late-stage remyelination.

[3] Remyelination in *Ccr2*$^{-/-}$ vs wildtype lesions by myelin protein expression: we compared the expression of the myelin protein MAG in lesions at 10 vs 21 dpl. *Ccr2*$^{-/-}$ mice consistently had decreased lesion MAG expression vs wildtype (**Fig.1M; new Fig.1O**), which was dissociated from the number of myelinated axons. Our results of more remyelination in *Ccr2*$^{-/-}$ is consistent with MAG global knockouts making excess myelin [*Li et al, 1994, Nature; Montag et al., 1994, Neuron*].

[4] Oligodendrocyte differentiation in *Ccr2*$^{-/-}$ vs wildtype lesions: We compared the impact of reduced classical monocytes on oligodendrocyte differentiation into mature cells (CC1+ OLIG2+) over the course of during remyelination. *Ccr2*$^{-/-}$ lesions had significantly reduced mature oligodendrocytes at 10 dpl, which then recovered to wildtype levels by 21 dpl (**new Fig.1J,K,P**).

[5] Oligodendrocyte differentiation in Wnt conditional knockout mouse lesions: We compared the impact of reduced Wnt release by monocytes on oligodendrocyte differentiation. We found no difference between conditional knockouts and floxed controls at remyelination onset (14 dpl), which was maintained by remyelination completion (21 dpl) (**new Fig.4D,E**), indicating that the impact of monocytes on oligodendrocyte differentiation is independent of Wnt release.
