# Supplementary material for: Monocyte-secreted Wnt reduces the efficiency of central nervous system remyelination
Source: PLoS Biol. 2025 Apr 15;23(4):e3003073. doi: 10.1371/journal.pbio.3003073 (PMC12052099; doi:10.1371/journal.pbio.3003073)
Supplement: Attachment — Submitted filename: Hill et al Plos Biol Response to Reviewers R2.pdf [file pbio.3003073.s010.pdf]

We are pleased to see that 3 of the 4 reviewers were satisfied by the significant changes we made with our revision. We agree that reviewer 3's concerns are easily addressed with textual changes and insertion of an additional graph to our manuscript. With regards to the editorial and reviewer comments, we have incorporated the suggestions into the revised manuscript, indicated in the main text with tracked changes, and outlined point-by-point below.

Editorial comments:

E1: Reviewer 3 has raised the concern that the study may underestimate the extent of monocyte differentiation - and we think that this is a reasonable concern. However, we would not require additional genetic fate mapping approaches to address this issue and think that it may be addressed with textual changes, and perhaps acknowledging this limitation, or toning down claims of monocyte identity. Reviewer 3 also notes a lack of enrichment of monocyte signature genes in the sorted monocyte RNA-seq dataset - and we wonder if this point could be addressed by simply presenting the FPKM data for a panel of established monocyte markers, similar to how you have presented microglia markers, in Fig 2a.
To address this issue we have 1) toned down claims of monocyte identity in the text, and 2) included a new graph which indicates enrichment of monocyte-signature genes (suggested by the reviewer) in the CD45^{hi} cell population (new SFig.3B).
We agree with the reviewer that it is challenging to definitively assess monocyte differentiation in lesions. We initially assessed protein expression of 2 standard markers commonly used to identify tissue macrophages, IBA1 and F4/80, via 2 complementary methods of immunofluorescence (in reporter mice) and flow cytometry (of CD45^{hi} cells); this indicated low/no macrophage marker expression. However, we acknowledge that a proportion of the lesion cells do not express the classical monocyte marker, Ly6C (SFig.3D-E), which could indicate initiation of macrophage differentiation (loss of monocyte marker, yet prior to macrophage marker expression). As such we have toned down our conclusion in the text to acknowledge the possibility of differentiation, or that this is a mixed population of differentiating/undifferentiated cells, as follows:
'Surprisingly, assessment of macrophage markers by flow cytometry of wildtype mice (F4/80 expression in CD11b^{+}CD45^{hi}Ly6C^{+} cells) (SFig.1F-G), or immunofluorescence analysis of Ccr2$^{RFP/+}$ mice (IBA1 expression in RFP^{+} cells) (SFig.1H-I) indicated that approximately 90% of the cells do not express typical macrophage maturation markers in remyelinating lesions. Considering the absence of expression of the monocyte marker Ly6C in a subset of the cells (SFig.1D-E), these findings could suggest that monocytes may be in the process of differentiating into macrophages, or that the population represents a mixture of undifferentiated and differentiating cells.'

E2: After some discussion within the team, we would like to propose an edit to the title to add a few more details from the study. If you agree (and if supported), we suggest you change the title to: "Monocyte-secreted Wnt reduces the efficiency of central nervous system remyelination"
We have revised the title as suggested.

E3: Please update the ethics statement in your methods section to include the full name(s) of the IACUC/ethics committee(s) that reviewed and approved the animal care and use protocol/permit/project license. Please also include an approval number.
We have updated the animal ethics committee and approval numbers as requested.

E4: Thank you for providing your RNA-seq data on GEO. Can you provide me with a reviewer token so that I can check that this meets our requirements? Also, please note that you will need to make that data publicly available before publication (although its fine to leave it private for now).
Here is the reviewer token for GSE121514: idyhcuaubfqllmb

E5: You may be aware of the PLOS Data Policy, which requires that all data be made available without restriction [...]. Note that we do not require all raw data. Rather, we ask that all individual quantitative observations that underlie the data summarized in the figures and results of your paper be made available...

We have uploaded all the quantitative data as a single supplementary excel sheet with the tabs indicating the figure panel. The data availability section has been updated to reference the supplemental information. The figure legends indicate that the underlying data is available as supplemental information.

E6: Please deposit the raw flow cytometry data to flow repository, or another publicly available repository.
Raw flow cytometry data has been uploaded to Flow Repository: http://flowrepository.org/id/FR-FCM-Z9ZJ .
We now indicate this in the data availability section.

Reviewer comments:

Reviewer 3:
3.1: The authors provided a detailed point-by-point rebuttal, but this reviewer is still not satisfied. Although I do not doubt that Wnt signaling from monocytes could play some role in regulating remyelination dynamics, the manuscript contains a large number of imprecisions, and several statements are either inaccurate, misleading or wrong. These are just a few examples of contradictory sentences:
"These findings suggest that when classical monocytes are reduced in remyelinating lesions, oligodendrocyte differentiation is impaired yet oligodendrocytes compensate by producing more myelin, suggesting that monocytes limit myelin production."
"Altogether, these findings reveal that although classical monocytes are required for the timely onset of oligodendrocyte differentiation and myelin protein expression during remyelination, they limit myelin production, thereby reducing remyelination efficiency."
I understand what the authors are trying to say, but the message is not clear at all.
We have revised these 2 sentences to improve clarity of our message.

3.2 "CNS lesion myeloid cells encompass resident populations (e.g. microglia) and infiltrating monocytes, which are considered to be indistinguishable from one another based on morphology and immunostaining for marker expression (12). Consequently, microglia and monocytes are often analysed as one population, with the respective contribution of each subset to remyelination being poorly understood." This is not true. Perhaps the authors wanted to say that MDMs can become very similar to microglia upon infiltrating the CNS parenchyma.
We have revised these sentences to refer to monocyte-derived macrophages.

3.3 "...our findings are in line with some of these genes having previously been shown to be expressed in monocytes albeit at a relatively lower level than in microglia (e.g. P2ry12, Tmem119), whereas Sall1 is never expressed by monocytes." Again, not true. Monocytes don't express P2ry12 and Tmem119. This is not what the literature shows. It looks like the authors do not distinguish between monocytes and MDM (monocyte-derived macrophages).
We have revised this sentence to refer to monocyte-derived macrophages.

3.4 "Surprisingly, assessment of macrophage markers by flow cytometry of wildtype mice (F4/80 expression in CD11b+CD45hiLy6C+ cells) (SFig.1F-G), or immunofluorescence analysis of Ccr2RFP/+ mice (IBA1 expression in RFP+ cells) (SFig.1H-I) indicated that approximately 90% of the cells remain undifferentiated as monocytes (F4/80-, IBA1-) in remyelinating lesions." The authors provide zero evidence of monocytes not differentiating in the lesion. Lack of F4/80 staining in CCR2+Ly6C+ monocytes is meaningless. Monocytes start upregulating macrophage markers along with downregulation of monocyte genes. Indeed, Fig. S3C suggests that monocytes are differentiating into macrophages.
We have revised our data interpretation to include the possibility of monocytes being in the process of differentiating into macrophages, or the population being a mixed population of undifferentiated and differentiating cells, due to the lack of expression of classical monocyte marker Ly6C along with the low to no expression of 2 standard pan-macrophage markers. We note however that the genes in SFig3C are known to

be expressed by monocytes as well as monocyte-derived macrophages; although they are upregulated with differentiation we have no comparative blood monocyte data to compare this, and the expression of these genes on their own in the lesion monocytes cannot therefore definitively indicate differentiation.

3.5 If the authors want to trace the fate of monocytes within the lesion, they should use a lineage tracing tool like Ms4a3-Cre or Ccr2-CreErt2 as suggested in the initial review. The authors claimed the following: "we note that this line has non-specific activity in other immune populations." I would like to know what the authors are referring to. Ms4a3-Cre recombines in BM GMPs, thus labeling monocytes (as well as MDMs) and neutrophils. These populations can be easily distinguished by flow cytometry, so I disagree with the concern about the specificity. Perhaps the authors chose not to perform this experiment. In this case, any statements pertaining to the lack of monocyte differentiation should be removed from the manuscript as it is not substantiated by experimental evidence.

We already used *Ccr2*-RFP/+ reporter mice to lineage trace classical monocytes infiltrating into remyelinating lesions, assessed by immunofluorescence and flow cytometry. The reviewer rightfully points out that *Ms4a3*-Cre recombines in cells other than monocytes, and this is what we were referring to in the context of generating a conditional knockout of the Porcupine gene. Although these different populations could be distinguished by flow cytometry, we would not be able to control for the impact of conditional knockout of Porcupine in neutrophils on remyelination. As outlined above, we have revised our sentence on monocyte differentiation to acknowledge that the cells could be in the process of maturing into macrophages based on expression, or lack of expression, of several markers.

3.6 Additionally, Fig.2C shows the top 50 DEGs between microglia and monocytes. This heatmap does not contain monocytes signature genes like Ly6c2, Ccr2, Ear2, Plac8, S100a4, Vim, Lyz2, Ms4a4c, just to mention a few. Only Cd44 is consistent with the monocyte signature. At the same time, this list of DEGs contain several neuronal genes (Map2, Tubb3, etc). Based on the data here provided, there is no reason to believe that monocyte differentiation is impaired in the demyelinating lesion; without even mentioning the tons of literature demonstrating the opposite.

We have now included a graph indicating the enrichment of monocyte-signature genes mentioned above, in lesion $CD45^{hi}$ cells (monocytes) vs $CD45^{lo}$ cells (microglia) (new SFig3.B). We reiterate that monocyte differentiation into macrophages has never been shown in *remyelinating* lesions, as prior studies in remyelinating models used reporters or markers expressed by both monocytes and monocyte-derived macrophages; nonetheless we have not pressed this point in the paper. We also highlight that *Map2* and *Tubb3* are known to be expressed in non-neuronal cells, e.g. oligodendrocytes, and are therefore not neuronal specific.

Reviewer 4:

4.1 The data presented in the new manuscript are vastly improved. I now think that the basis of the paper is much more solid, although much of the data are not definitive and there are many open questions, the readers now have enough information to assess it for themselves. I would still require that information regarding the cell numbers for sequencing be included, as is described in the rebuttal: 'the average cell yield for sorted microglia was 5777 per mouse, and the average cell yield of sorted monocytes was 38 per mouse.....we sequenced an average of 4 ng of RNA for microglia and 1.6 ng of RNA from monocytes.' This is important to know for those wanting to assess the quality but also repeat the experiment should they wish.

We have now included this information in the Methods section of the paper.
